# Inhibition and Mechanism of Protein Nonenzymatic Glycation by *Lactobacillus fermentum*

**DOI:** 10.3390/foods13081183

**Published:** 2024-04-12

**Authors:** Qin Li, Ke Xiao, Chi Yi, Fan Yu, Wenyue Wang, Junhui Rao, Menglin Liu, Lin Zhang, Yang Mu, Chao Wang, Qian Wu, Dongsheng Li, Mengzhou Zhou

**Affiliations:** Key Laboratory of Fermentation Engineering (Ministry of Education), Hubei Key Laboratoy of Industrial Microbiology, National “111” Center for Cellular Regulation and Molecular Pharmaceutics, Hubei Research Center of Food Fermentation Engineering and Technology, Hubei University of Technology, Wuhan 430068, China; karenlovely1026@163.com (Q.L.); 73186410629r@gmail.com (K.X.); vumpine@163.com (C.Y.); y1290378292@163.com (F.Y.); wangwenyue1228@163.com (W.W.); raojunhui123@gmail.com (J.R.); menglin5@ualberta.ca (M.L.); Linnnnnn97@gmail.com (L.Z.); 20140017@hbut.edu.cn (Y.M.); wangchaohugong@sina.com (C.W.); qianwill2007@163.com (Q.W.); dongshengli86@163.com (D.L.)

**Keywords:** glycation, *Lactobacillus*, secondary structures, antioxidation, advanced glycation end-products

## Abstract

*Lactobacillus fermentum* (*L. fermentum*) was first evaluated as a potential advanced glycation end-product (AGE) formation inhibitor by establishing a bovine serum albumin (BSA) + glucose (glu) glycation model in the present study. The results showed that the highest inhibition rates of pentosidine and total fluorescent AGEs by *L. fermentum* were approximately 51.67% and 77.22%, respectively, which were higher than that of aminoguanidine (AG). Mechanistic analysis showed that *L. fermentum* could capture methylglyoxal and glyoxal, inhibit carbonyl and sulfhydryl oxidation, reduce the binding of glucose and amino groups, increase total phenolic content and antioxidant activity, and release intracellular substances to scavenge free radicals; these abilities were the basis of the antiglycation mechanism of *L. fermentum*. In addition, *L. fermentum* significantly prevented conformational changes in proteins during glycation, reduced protein cross-linking by 35.67%, and protected the intrinsic fluorophore. Therefore, the inhibition of *L. fermentum* on glycation mainly occurs through antioxidation, the capture of dicarbonyl compounds, and the protection of the BSA structure. These findings collectively suggest that *Lactobacillus* is an inhibitor of protein glycation and AGE formation and has the potential for nutraceutical applications.

## 1. Introduction

Since Monnier proposed in 1981 that nonenzymatic glycation of proteins in vivo may be related to aging and related diseases [1], the “non-enzymatic glycation aging hypothesis” [2] has revealed that nonenzymatic glycation reaction products are one of the biomarkers of aging [3]. The free amino groups in the protein structure and the carbonyl groups of reducing sugars undergo complex reactions, such as condensation, rearrangement, degradation, and oxidation, under nonenzymatic conditions to generate highly irreversible AGEs [4]. The formation of these AGEs may not only change the secondary structure of proteins, but also activate (NF) – kappaB (NF-Κb) by binding to its receptors, aggravating the oxidative stress and inflammatory response of the body, and their accumulation in the cells and intercellular matrix leads to pathological reactions in the tissues and organs [5,6]. Corzo-Martínez et al. [7] found that after ingesting glycated BSA, patients with ulcerative colitis showed decreased levels of the dominant beneficial bacteria in their intestinal flora, while the levels of the harmful bacteria increased. Therefore, AGEs have pathogenic effects on some chronic diseases, such as diabetes and atherosclerosis, making the development of AGE inhibitors a research hotspot.

Aminoguanidine effectively inhibits the formation of AGEs by eliminating reducing carbonyl groups and reducing the formation of α-dicarbonyl compounds [8]. However, high doses of aminoguanidine caused gastrointestinal side effects and toxicity in clinical studies [9]. In contrast, some safer and beneficial plant extracts extensively studied, such as myricetin polyphenols [10], bamboo leaf flavonoids [11], and lotus leaf extracts [12], have proven to be natural inhibitors of protein glycation or the formation of AGEs owing to their antioxidant properties, scavenging free radical activity, chelating metal ion activity, capturing active carbonyl compounds, and protecting protein glycation sites. Although these natural inhibitors showed good antiglycation activity, they have not yet been widely used in clinical practice due to many uncertain influencing factors. As reported by Yang et al. [13], low concentrations (12.5–100 µM) of (+)-catechin inhibited carboxymethyl lysine (CML) formation, whereas high concentrations (200–800 µM) of (+)-catechin did not. Catechins inhibit methylglyoxal (MGO)-induced CML formation in the presence of catalase, whereas high concentrations of (+)-catechins increase H_2_O_2_ formation and increase catalase consumption. It is difficult for the human body to ensure the effective content of the abovementioned natural inhibitors in the daily diet; excessive intake of plant compounds and flavonoid plant compounds may have a negative effect on the body, resulting in side effects, such as dermatitis, hemolytic anemia, and even liver failure [14]. In addition, catechins in green tea are very unstable under both the processing environment and the gastrointestinal tract conditions of the body [15], and their chemical stability and bioavailability are affected by the processing and formulation conditions [16]. Given the abovementioned problems of antiglycation inhibitors, it is necessary to explore a more reliable and safer dietary therapy to inhibit the nonenzymatic glycation of proteins and the formation of AGEs in clinical practice.

According to the definition by the FAO/WHO, probiotics refer to a class of live microorganisms that are beneficial to the health of the host after sufficient intake [17], mainly distributed in the genera *Lactobacillus* and *Bifidobacterium* [18]. *Lactobacillus* spp., including *Lactobacillus fermentum* and *L. plantarum*, play an important role in regulating the physiological functions of the host and maintaining the health of the host. Probiotic functions that have been confirmed include relieving lactose intolerance, promoting nutrient absorption, inhibiting intestinal pathogens, preventing and treating peptic ulcers, and improving the immune ability of the body to maintain intestinal microecological balance [19]. Currently, *Lactobacillus* spp. are widely used in food and medical and health products. According to relevant literature reports, *Lactobacillus* spp. together with tea polyphenols can improve the blood lipid balance of the intestinal system and the overall health of animals [20]. Biscuits made from buckwheat flour fermented with *Lactobacillus* spp. had higher antioxidant activity and bioavailability in the gastrointestinal tract of the body [21]. Edible cyanobacteria and microalgae fermented by *Lactobacillus* spp. showed strong free radical scavenging ability and antiglycation activity [22]. These results showed that *Lactobacillus* spp. may be an ideal candidate for natural AGE inhibitors. However, to our best knowledge, *Lactobacillus* as an AGE inhibitor and its underlying mechanisms have not yet been reported.

Therefore, this study aimed to measure the formation of glycation products, changes in protein structure, and functional groups by simulating glycation models in vitro and using methods, such as fluorescence spectroscopy, Fourier transform infrared spectroscopy (FTIR), and liquid chromatography, to systematically explore the inhibitory potential and mechanism of a laboratory-derived *L. fermentum* on nonenzymatic glycation of proteins.

## 2. Material and Methods

### 2.1. Materials and Chemicals

BSA and gallic acid were provided by the China National Pharmaceutical Group. Glu, FeSO_4_, 0.01% H_2_O_2_, K_2_S_2_O_8_, Folin phenol reagent, potassium ferricyanide, ethylenediaminetetraacetic acid disodium salt (EDTA), and trichloroacetic acid (TCA) were obtained from Sinopharm Chemical Reagent Co. (Shanghai, China). Lactic acid bacteria (LAB) preserved in a laboratory, arginine (Arg), glyoxal (GO) (40% aqueous solution), Methylglyoxal (40% aqueous solution), aminoguanidine, diammonium salt (ABTS), 1-anilino-8-naphthalene sulfonate (ANS), 1,1-diphenyl-2-picrylhy-drazyl (DPPH), and ortho-phthalaldehyde (OPA) were purchased from Macklin Biochemical Co. (Shanghai, China). In addition, ο-phenylenediamine (ο-PDA), nitroblue tetrazolium (NBT), 2,4-dinitrophenylhydrazine (2,4-DNPH), 1,10-phenanthroline, and 5,5′-dithiobis (2-nitrobenzoic acid) (DTNB) were purchased from Aladdin (Shanghai, China). All reagents were of analytical grade.

### 2.2. Preparation of Complete Cell Suspension of LAB

*L. fermentum* frozen in a glycerol tube were inoculated in liquid MRS medium for recovery, cultured at 37 °C for 18 h, and activated for two generations for use in the experiment. The fermentation broth was cultured for 18 h and centrifuged at 8000× *g* for 5 min; the bacteria were collected and then washed with PBS buffer (0.2 M, pH 7.4) three times to wash off the medium. The active *L. fermentum* content of the above strains is more than 10^8^ CFU/g.

### 2.3. The Antiglycation Capacity of L. fermentum in a BSA + Glu Model

The antiglycation assay in a BSA + Glu model was used to evaluate the inhibitory effect of LAB on AGE formation, according to the method of Peng et al. [23]. In brief, 15 mg/mL BSA in PBS (0.2 M, pH 7.4) with 150 mg/mL of glucose was filtered through a 0.22-µm Millipore filter into sterile capped vials and incubated at 37 °C in the dark for 2, 4, 6, 8, and 10 days. *L. fermentum* was inoculated in BSA + Glu, and the final concentration was 1.2 mg/mL, which was regarded as the experimental group. The same concentration of aminoguanidine was added as the positive control group and the glucose solution was replaced with PBS in the blank control. After incubation was completed, the bacteria and supernatant obtained through centrifugation were preserved at −20 °C for further analysis.

#### 2.3.1. Fluorescent AGEs

The formation of total fluorescent AGEs and pentosidine in each reaction solution was measured using a Hitachi F-4700 fluorescence spectrophotometer (F-4700; Hitachi, Ltd., Tokyo, Japan) at excitation/emission wavelengths of 370 nm/440 nm and 335 nm/385 nm, respectively, with both excitation and emission bandwidths of 5 nm [10]. The percentage inhibition of AGEs or pentosidine formation was calculated using the following equation:

AGE inhibition (%) = [1 − (F_sample_ − F_sample blank_)/(F_control_ − F_control blank_)] × 100%

In the equation, F_sample_ is the fluorescence value of AG + BSA + Glu or LAB + BSA + Glu, Fsample blank is the fluorescence value of AG + BSA + PBS or LAB + BSA + PBS, Fcontrol is the fluorescence value of BSA + Glu, and Fcontrol blank is the fluorescence value of BSA + PBS.

### 2.4. Measurement of pH and Glucose Concentration

The pH was determined using a digital pH meter. Glucose was analyzed using the 3,5-dinitrosalicylic acid (DNS) colorimetric method [24]. DNS colorimetry is a common method for the determination of reducing sugars. DNS was used as a chromogenic agent and glucose standard solution as a reference. The amino compounds formed by reducing sugars in alkaline solution had the maximum absorption at 540 nm wavelength.

### 2.5. Amadori Product and α-Dicarbonyl Compounds

Fructosamine was a typical Amadori product during glycation, and it was quantified using an NBT assay according to the method of Zhang et al. [25] glyoxal and Methylglyoxal were determined using precolumn derivatization high-performance liquid chromatography. OPA is a commonly used catcher and derivative of α-dicarbonyl compounds. The derivation of α-dicarbonyl compounds by OPA can produce stable quinoxaline derivatives. Among them, the derivation of glyoxal to quinoxaline (Q) and Methylglyoxal to 2-methylquinoxaline (2-MQ) can be detected quickly and accurately using high-performance liquid chromatography (HPLC). An HPLC system (Agilent 1260 Infinity, Santa Clara, CA, USA) was connected to a Unitary C18 column (150 × 4.6 mm, i.d., 5-mm particles), and a UV absorbance detector for the analysis of Q and 2-MQ using the method described by Song et al. [26].

### 2.6. Determination of Functional Group Changes

#### 2.6.1. Protein-Bound Carbonyl

Protein-bound carbonyl is the main indicator used to measure protein oxidative damage, and its content indicates the degree of protein oxidative damage. The content of protein-bound carbonyl was determined using the method described by Eze et al. [27].

One hundred microliters of the above sample solutions were mixed with 400 µL 2,4-DNPH (10 mM); the mixed solution was reacted at 37 °C for 60 min in the dark, and vortexed once every 10 min during the period. Subsequently, 500 µL of trichloroacetic acid (20%; *w*/*v*) was added to precipitate the protein hydrazone derivative, and the solution was put on ice for 10 min to precipitate the protein. This was further centrifuged at 4 °C and 10,000× *g* for 10 min; the isolated protein precipitate was washed with 1 mL ethanol/ethyl acetate (1:1; *v*/*v*) mixed solution, and the above steps were repeated three times. The resulting protein precipitate was dissolved with 1.25 mL guanidine hydrochloride (6 M) and placed in a water bath at 37 °C for 15 min. The absorbance was measured at 370 nm using a UV–Vis spectrophotometer. The carbonyl concentration was calculated using a molar extinction coefficient of 22 mmol/(L·cm), and the carbonyl content was expressed as µmol/mg protein.

#### 2.6.2. Sulfhydryl (SH) Groups

Using the method of Sirichai et al. [28] with a slight modification, the protein concentration in the sample solution was adjusted to 5 mg/mL with 0.02 M PBS (pH 6.0, containing 0.6 mol/L NaCl). After mixing, 1 mL of the diluent was transferred to another centrifuge tube, and 9 mL of PBS (0.05 M, pH 7.2, containing 0.6 mol/L NaCl, 6 mmol/L EDTA-2Na, and 8 M urea) was added to it, and mixed well. Next, 3 mL of the above mixture and 0.5 mL of 0.1% DTNB were added to another clean test tube, and the solution was reacted at 40 °C for 25 min in the dark. After the reaction, the absorbance of the solution at 412 nm was measured, and the SH group was calculated using the molar extinction coefficient of 13,600 M^−1^cm^−1^ and according to the following formula:

SH group (nmol/mg protein) = 75.53 × A_412_ nm/C

In the formula, C is the protein concentration (mg/mL) and A_412_ nm is the absorbance of the solution to be tested at 412 nm.

#### 2.6.3. Free Amino Groups

The free amino groups content of the sample was measured using the OPA method, and the glycation degree of the sample was determined indirectly [29]. The preparation steps for the OPA reagent are described below. Briefly, 40 mg OPA was dissolved in a mixture containing 1 mL of 95% methanol, 25 mL of sodium tetraborate buffer (containing 100 µL β-mercaptoethanol), and 2.5 mL of 20% (*w*/*v*) SDS solution. The final mixture was diluted to 50 mL with deionized water, which was used as the OPA working solution. Next, 100 µL of the sample solution was mixed with 2 mL of OPA working solution, reacted in a water bath at 40 °C for 3 min, and then the absorbance was measured at 340 nm using a UV–Vis spectrophotometer immediately. The relative percentage of free amino groups in the glycation process was calculated as follows:

Free amino content (%) = (A_1_ − A_2_ − A_3_)/(A_N-BSA_ − A_2_) × 100%

In the formula, A_1_ is the absorbance value of the sample, A_2_ is the absorbance value of the reaction between the double-distilled water instead of the sample and the OPA working solution, A_3_ is the absorbance value of the mixed solution of 100 µL sample and 2 mL double-distilled water, and A_N-BSA_ is the absorbance value of the reaction between the N-BSA control group and OPA working solution.

### 2.7. Evaluation of Structural Changes

#### 2.7.1. Intrinsic Tryptophan Fluorescence

The tryptophan characteristic fluorescence was induced using two tryptophan residues in the unfolded structure of BSA, and its value was influenced by protein fibrillation and its conformation or folding. The tryptophan fluorescence emission spectra of the samples were characterized under the excitation wavelength of 295 nm and the emission wavelength in the range of 315–415 nm; the slit width and the interval time were 2 nm and 0.5 s, respectively [30,31].

#### 2.7.2. ANS-Fluorescence

The effect of LAB on the surface hydrophobicity of glycated BSA was monitored using the ANS fluorescent probe method [32]. ANS, a fluorescent dye used for protein research, has been widely used to monitor exposed protein hydrophobic plaques and their folding intermediates, protein misfolding, and the presence of surface hydrophobic groups. Upon binding of ANS to nonpolar regions of the protein surface or membrane, mobility is restricted resulting in a blue shift of the fluorescence emission maxima and an increase in fluorescence intensity. An appropriate amount of ANS was weighed and completely dissolved in dimethylformamide solution to prepare ANS with a concentration of 30 µmol/mL. The sample and ANS solution were mixed in equal volumes and reacted at 25 °C for 30 min in the dark. Scanning recordings were performed at a fluorescence excitation wavelength of 380 nm and emission wavelengths in the range of 390–650 nm, with excitation and emission slit widths of 5 nm. The resulting measured spectra were adjusted for blank values.

#### 2.7.3. SDS-PAGE

The method of Zhang et al. [33] was used with appropriate modifications. After appropriate dilution of the sample solution, 16 µL was mixed with 4 µL of 5 × loading buffer, heated at 100 °C for 10 min, and then centrifuged at 8000× *g* for 1 min. Ten microliters of the mixture was injected into a gel lane consisting of 5% stacking gel and 10% separating gel for electrophoresis.

#### 2.7.4. Fourier Transform Infrared Spectroscopy (FTIR) Spectra

After incubation, the sample solution was placed into an 8000 Da dialysis bag and fully dialyzed with PBS for 24 h to remove unbound glucose. The collected sample solution was freeze-dried into a dry piece of uniform thickness, which was measured using multiple attenuated total reflection (ATR), and the dry piece was placed on the ATR accessory for scanning. The infrared spectrum was recorded using OMNIC software 8.0; the measurement range was 4000–400 cm^−1^, the wavelength accuracy was 0.01 cm^−1^, the resolution was 4 cm^−1^, the number of scans was 32, and the ambient temperature was 25 °C [34].

The peaks at 1600–1700 cm^−1^ were fitted using the Fourier deconvolution, second derivative derivation, and Gaussian curve, and the relative content of each secondary structure of the protein was calculated using the peak area according to the fitting results [35].

### 2.8. Determination and Verification of Trapping Methylglyoxal/Glyoxal

#### 2.8.1. Methylglyoxal/Glyoxal Trapping Ability Determination

The concentration of methylglyoxal or glyoxal solution was diluted with PBS to 0.6 mM or 0.8 mM, respectively [36], and then mixed with an equal volume of bacterial solution. The contents of methylglyoxal and glyoxal were measured after incubation at 37 °C for 1 day. The *L. fermentans* capture capacity was calculated according to the following formula:

Clearance rate (%) = (1 − C_1_/C_0_) × 100%.

In the formula, C_0_ is the methylglyoxal/glyoxal concentration measured at the beginning and C_1_ is the methylglyoxal/glyoxal concentration after one day of culture.

#### 2.8.2. The Antiglycative Capacity of *L. fermentum* in Methylglyoxal + BSA/Methylglyoxal + Arg Models

The methylglyoxal + BSA and methylglyoxal + Arg models were used to evaluate the inhibitory effect of LAB on the intermediate stages of the nonenzymatic glycation of proteins [36]. The methylglyoxal +BSA model consisted of equal volumes of 45 mg/mL BSA, 6 mM methylglyoxal, and bacterial suspension. PBS, instead of the bacterial suspension, was used as a blank control, and PBS, instead of the methylglyoxal solution, was used as a control. In the methylglyoxal + Arg model, Arg was used instead of BSA, and the other preparation methods remained unchanged. After incubating at 37 °C for 48 h in the dark, the formation of total fluorescent AGEs and pentosidine in each reaction solution was measured at excitation/emission wavelengths of 370 nm/440 nm and 335 nm/385 nm, respectively.

### 2.9. Antioxidant Activity Assays

#### 2.9.1. DPPH (2,2,-Diphenyl-1-picrylhydrazyl) Radical Scavenging Assay

Using the method of Li et al. [37] with a slight modification, 0.2 mM DPPH solution was prepared with absolute ethanol; the sample and DPPH solutions were mixed with equal volumes and shaken, and then reacted at room temperature for 30 min in the dark. Then, the absorbance value was measured at a wavelength of 517 nm and recorded as A_1_. An equal volume of the sample solution was mixed with absolute ethanol and shaken, and the absorbance value was measured under the same conditions as above and recorded as A_2_. The same volume of DPPH solution was mixed with pure water and shaken well, and the absorbance value was measured under the same conditions as above and recorded as A_3_. The DPPH free radical-scavenging rate formula is shown below:

DPPH free radical-scavenging rate (%) = [1 − (A_1_ − A_2_)/A_3_] × 100%.

#### 2.9.2. Hydroxyl Radical-Scavenging Rate

Using the method of Zhang et al. [38] with a slight modification, 1 mL of sample solution, 1,10-phenanthroline (0.75 mM), FeSO_4_ solution (0.75 mM), and 0.01% H_2_O_2_ solution were added to a test tube, followed by 1.5 mL of PBS (pH 7.4, 0.15 M), and fully mixed and reacted at room temperature for 1 h. The absorbance (denoted as A_1_) was measured at A_536_ nm; the absorbance measured with pure water instead of the sample solution was recorded as A_2_, and the absorbance measured with pure water instead of the sample solution and 0.01% H_2_O_2_ solution was recorded as A_3_. After recording the data, the hydroxyl radical-scavenging rate was calculated using the following formula:

Hydroxyl radical-scavenging rate (%) = (A_1_ − A_2_)/(A_3_ − A_2_) × 100%.

#### 2.9.3. ABTS Radical Cation-Scavenging Activity

Using the method of Du et al. [39] with a slight modification, the ABTS reaction solution was prepared using an equal volume of ABTS solution (7.4 mM) mixed with K_2_S_2_O_8_ solution (2.6 mM); the mixed solution was fully reacted for 12 h at 25 °C in the dark, and then the above reaction solution was diluted with absolute ethanol until the absorbance range was 0.7 ± 0.02 and ready for use in the experiment. The sample solution and the ABTS reaction solution were fully mixed in a volume ratio of 1:5, and the absorbance of the mixed solution at 734 nm was measured immediately, which was recorded as A_2_. The ABTS reaction solution was mixed with 60% ethanol solution in the same proportion to measure the absorbance and recorded as A_1_. Finally, the free radical-scavenging rate of ABTS was calculated according to the following formula:

ABTS free radical-scavenging rate (%) = (A_1_ − A_2_)/A_1_ × 100%.

#### 2.9.4. Total Reducing Power Assay

Using the method of Suktham et al. [40] with a slight modification, 1 mL of the sample solution, 2.5 mL of PBS (pH = 6.6, 0.2 M), and 1 mL of 1% potassium ferricyanide solution were fully shaken and mixed evenly, and then reacted at 50 °C for 20 min. After the reaction, the solution was cooled to room temperature, 2.5 mL of 10% trichloroacetic acid was added to the solution, mixed well, and then centrifuged at 5000 r/min for 10 min. After centrifugation, 5 mL of the supernatant was mixed with 1 mL of FeCl_3_ solution (1%, *w*/*v*) and 5 mL of pure water. The absorbance of the mixture was measured at 700 nm and recorded as A_1_ and then FeCl_3_ solution was replaced with pure water and the other measurement conditions were kept consistent; the measured absorbance value was recorded as A_2_. The formula used for calculation is as follows:

Total reducing power rate = A_1_ − A_2_.

### 2.10. Total Phenolic Content

Total phenolic content was determined using the Folin phenol method [29]. Briefly, 1 mL of the sample solution and 0.5 mL of Folin phenol reagent were mixed evenly and allowed to react for 5 min. Next, 1.5 mL of 7.5% Na_2_CO_3_ solution was added, the volume was adjusted to 6 mL with ultrapure water, shaken well, and reacted in the dark for 30 min at room temperature. After that, the absorbance was measured at 760 nm. The 2, 4, 6, 8, and 10 µg/mL of gallic acid standard solution were used to measure the absorbance value according to the above experimental procedure, and the gallic acid standard curve (0–10 µg/mL) was drawn to obtain the gallic acid equivalent (GAE) of micrograms per mL of the sample solution.

### 2.11. Determination of the Total Viable Count (TVC) and Morphological Changes of L. fermentum

TVC of *L. fermentum* in the BSA + Glu model was determined. The centrifuged bacteria were appropriately diluted with sterilized physiological saline, then poured into MRS solid culture plates and spread evenly. After anaerobic culture at 37 °C for 48 h, the colonies were counted, and the viable bacteria counts were expressed as per mL of sample (CFU/mL). The morphology of the bacteria was observed using a scanning electron microscope.

### 2.12. The Anti-AGE Activity of Intracellular Substances

Referring to 2.2, the cell wall of the bacterial suspension was disrupted using a cell disruptor and then centrifuged to obtain intracellular fluid. The viable bacteria solution, 45 mg/mL BSA and 450 mg/mL glucose were mixed in equal volumes as the viable bacteria group; the viable bacteria solution was replaced with intracellular fluid as the intracellular fluid group. The glucose solution in each group was replaced with PBS as the corresponding control group. The sample solutions were incubated at 37 °C in the dark for 48 h, and the fluorescence values of each solution were measured, and their AGE inhibition rates were calculated, as described in Section 2.3.1.

### 2.13. Preparation of Active Components of L. fermentum

LAB were incubated in a BSA + Glu system for 48 h and then centrifuged at 4 °C and 8000× *g* for 10 min, after which the supernatant was filtered using a 0.22-μm Millipore filter; this was the fermentation supernatant (FS). Intact LAB cells were crushed in an ultrasonic ice bath (300 w, working time 5 s, interval 5 s, 30 min) and centrifuged, and then the supernatant was filtered using a Millipore filter to obtain intracellular extracts (IE). The precipitation was dissolved in 10% TCA, stirred at 4 °C for 16 h, and centrifuged, after which the supernatant and precipitate were collected. Among them, the supernatant and ethanol were mixed using a volume ratio of 1:2, alcoholized at 4 °C for 24 h, and centrifuged. The precipitation was washed with sterile water three times, and finally teichoic acid (TA) was collected. In addition, the precipitates obtained after TCA treatment were suspended in 10% TCA, stirred in a boiling water bath for 10 min, cooled, and centrifuged. Next, the precipitates were washed with sterile water three times. Finally, peptidoglycan (PG) was collected.

As described in Section 2.3, the fermentation supernatant, intracellular matter, peptidoglycan, and teichoic acid were prepared using the volume of LAB in the experimental group LAB + BSA + Glu. To explore the effects of these four active ingredients in the BSA + Glu glycation system, combined with the results of the previous experiment, the four experimental groups were incubated for 5 days, and the culture medium was collected for subsequent studies.

### 2.14. Statistical Analysis

Origin 8.0 software was used to conduct single and multiple variance analyses of each group of data. The results are expressed as the mean plus or minus the standard deviation (mean ± SD). The significant level was expressed as *p* < 0.05 if there were differences between the groups.

## 3. Results and Discussion

### 3.1. Inhibition of Fluorescent AGE Formation

**Figure 1:**
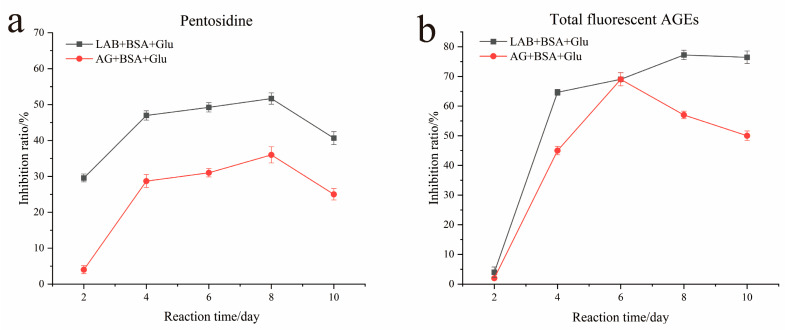
Inhibitory effect of pentosidine (**a**) and total fluorescent AGEs (**b**) by *L. fermentum* in the BSA + Glu system and AG+ BSA + Glu system.

Protein nonenzymatic glycation undergoes a series of complex and slow reactions and finally forms fluorescent AGEs with different structures. Therefore, various fluorescent AGEs can be detected using different excitation wavelengths. As shown in Figure 1a, the inhibition rate of *L. fermentum* to pentosidine in the glycation reaction was approximately 50%, while the inhibition rate of aminoguanidine to pentosidine was approximately 36%. The total fluorescent AGE content of LAB + BSA + Glu decreased by 1.35- and 1.5-fold compared with that of AG + BSA + Glu on day 8 and day 10, respectively (Figure 1b). The results showed that *L. fermentum* exhibited a stable inhibitory effect on both pentosidine and total fluorescent AGEs, and the inhibition rate was significantly higher than that of the positive control (*p* < 0.05). In a previous study, *L. fermentum* was found to significantly reduce the content of AGEs in fermented food (*p* < 0.05), and the mechanism remains unclear [41,42]. In the present study, a BSA + Glu nonenzymatic glycation model was constructed to further explore the mechanism of AGE inhibition by *L. fermentum*.

### 3.2. Effect on the Physical and Chemical Properties of the System

**Figure 2:**
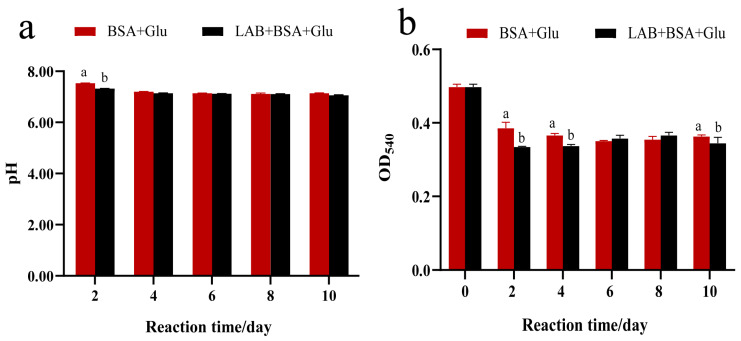
Determination of pH (**a**) and glucose concentration (**b**) in the glycation reaction system. Significance is at the 0.05 level (*p* < 0.05). Different letters indicated significant differences (*p* < 0.05).

For an in vitro glycation model, both pH and substrate concentration affect the generation of AGEs. AGEs are usually formed in a neutral pH environment, rather than in alkaline and acidic conditions. In an alkaline environment, Amadori compounds were more converted into nonfluorescent substances such as CML, which led to a decrease in their fluorescence value with increasing pH. Acidic conditions can inhibit the formation of AGEs; thus, foods soaked in vinegar and lemon show a significant reduction in AGEs [43]. Compared with the negative control, in Figure 2a, the addition of *L. fermentum* did not change the pH of the system, which was stable in a neutral environment during the incubation period. Glucose is one of the important nutrients of *L. fermentum* and an important substrate for protein glycation; its content affected the production of AGEs. In the early stage of the glycation reaction, a large amount of glucose was auto-oxidized to form dicarbonyl compounds, which led to a decrease in its content. Comparing the glucose content of the *L. fermentum* group with that of the negative control on the 2nd, 4th, and 10th days, glucose was consumed by *L. fermentum* (Figure 2b), which decreased by approximately 15.36%, 6.31%, and 7.48%, respectively. Of note, the inhibition rate of AGEs by *L. fermentum* was over 70%, which was far higher than the decreased inhibition rate of glucose indicating that the consumption of glucose was not the main reason for the inhibition of AGE formation.

### 3.3. Inhibition of Early and Mid-Stage Glycation Products

Early glycation reaction products are also important for promoting the formation of AGEs, including Amadori products undergoing oxidation or nonoxidation, which rearrange to form AGEs. Active carbonyl compounds cross-link with lysine or arginine on proteins to form AGEs. Therefore, it is also important to inhibit the formation of precursors for the formation of AGEs.

**Figure 3:**
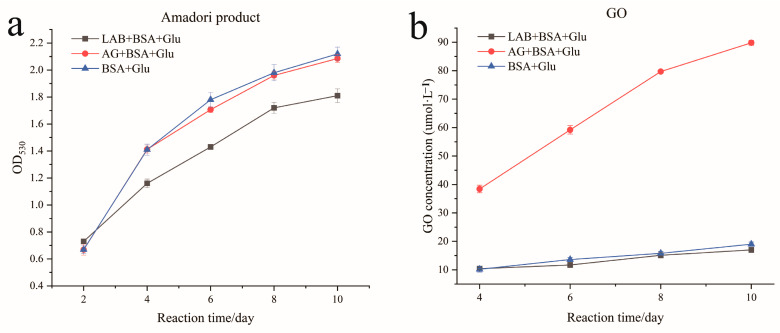
Inhibition of (**a**) Amadori products and (**b**) dicarbonyl compounds.

Amadori products are the first stage products of the nonenzymatic glycation of protein, which are formed by combining reducing sugars with free amino groups on proteins. As shown in Figure 3a, during the cultivation process, the content of Amadori products in the negative control increased continuously. Compared with the control group, the production of Amadori products reduced significantly in the *L. fermentum* group (*p* < 0.05) and decreased by 15% on the 10th day, which may be due to reduced glucose binding to free amino groups or inhibition of BSA and glucose to generate the Schiff base. However, there was no significant difference between the positive and negative controls, indicating that aminoguanidine could not inhibit the production of Amadori products.

The second stage of the glycation reaction is the formation of dicarbonyl compounds, mainly glyoxal and methylglyoxal. As shown in Figure 3b, during the culture of the BSA + Glu system, no methylglyoxal was detected in the *L. fermentum* and negative control groups, and the detected glyoxal content was low. The glyoxal content of BSA + Glu was approximately 10.2%, showing a steady upward trend, and approximately 19% by the 10th day. This result may have been caused by the incubation time, temperature, and other conditions of the glycation model. It was observed that the production of glyoxal was inhibited by *L. fermentum*, and after the 10 days reaction time, reducing glyoxal production in BSA + Glu by an average of 2%. Although glyoxal may have been captured by *L. fermentum*, the content of glyoxal was higher in the aminoguanidine group than in the negative control group, which was consistent with the results of Zhao et al. [44] on the inhibition of glycation by black chokeberry.

### 3.4. Analysis of Functional Group Changes

**Figure 4:**
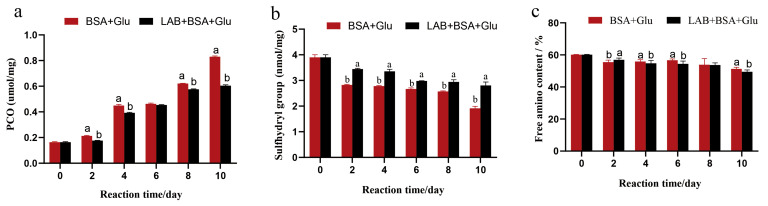
The effect of *L. fermentum* on (**a**) protein carbonyl, (**b**) sulfhydryl group, and (**c**) free amino content in the BSA + Glu system. Significance is at the 0.05 level (*p* < 0.05). Different letters indicated significant differences (*p* < 0.05).

The formation of protein carbonylation is an important marker of protein oxidation, and this process is accompanied with oxidative stress and reactive oxygen species (superoxide anions and hydroxyl radicals) generation. The protein-bound carbonyl content of BSA gradually increased during the glycation process. The protein-bound carbonyl content of BSA + Glu samples reached the highest value of 0.83 µmol/mg on day 10, which was significantly higher than that of the N-BSA group (0.16 µmol/mg). In the presence of *L. fermentum*, the protein-bound carbonyl content reduced; compared with the BSA + Glu sample group, the production of the protein-bound carbonyl group in the *L. fermentum* group decreased significantly (*p* < 0.05) (Figure 4a). The results showed that *L. fermentum* could inhibit the formation of protein carbonylation during protein glycation. This was consistent with the results of the inhibition of protein glycation by the antiglycation component of Salvia officinalis L. studied by Raâfet et al. [45], suggesting that *L. fermentum* may also play a role in scavenging reactive oxygen species and free radicals in the glycation reaction.

The sulfhydryl group in the glycation reaction is more sensitive and easier to be oxidized by hydroxyl radicals to form intermolecular or intramolecular disulfide bonds, resulting in a decrease in its number. Therefore, the sulfhydryl group content is also an important indicator of protein oxidation. As shown in Figure 4b, the sulfhydryl content of the BSA + Glu group on day 10 was 1.8 nmol/mg, which was significantly lower than that of the N-BSA group (4 nmol/mg) (*p* < 0.05), and after the addition of *L. fermentum*, the sulfhydryl content was 2.7 nmol/mg. Compared with the BSA + Glu group, the addition of *L. fermentum* significantly inhibited the decrease in the sulfhydryl content in the glycation reaction (*p* < 0.05). These results show that the addition of *L. fermentum* also inhibited the consumption of sulfhydryl groups, and the same results were found in the inhibition of cyanidin against glucose- and methylglyoxal-induced protein glycation [46]. This further confirmed the presence of antioxidant and protective proteins in the *L. fermentum* saccharification system.

The free amino group in the molecular structure of BSA is the active group required for the generation of fluorescent AGEs. During the oxidation process, the -NH- and -NH2 of the amino acid side chain are easily attacked by free radicals and can directly participate in the formation of carbonyl groups. The carbonyl group formed can further bond covalently with -NH2, resulting in the reduction in the free amino group content after the glycation reaction. As shown in Figure 4c, the relative content of free amino groups in the N-BSA group was 60.2%, and the relative content of free amino groups in the BSA + Glu group was 51.6% after 10 days of glycation system reaction. The attack of hydroxyl radicals decreased the content of free amino groups, which was significantly lower than that in the N-BSA group (*p* < 0.05). After adding *L. fermentum*, the relative content of free amino groups was 48.3%, which was not significantly different from that of the BSA + Glu group. Therefore, although *L. fermentum* may not inhibit the loss of free amino groups during glycation, it can inhibit the combination of glucose and free amino groups to generate Amadori products. Similar to the results of the inhibitory mechanism of quercetin on AGE formation described by Cao et al. [47], it is possible that *L. fermentum* may cause its content to decrease by combining with amino groups.

### 3.5. Capture Ability for Methylglyoxal/Glyoxal

**Figure 5:**
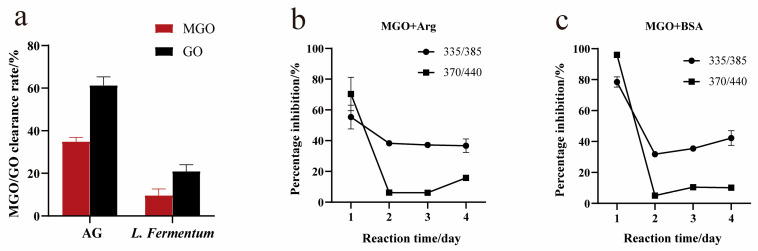
(**a**) Methylglyoxal (MGO) and glyoxal (GO) trapping capacity of *L. fermentum* in 24 h, (**b**,**c**) inhibition of pentosidine and AGEs in the MGO + BSA and MGO + Arg models. Among them, in (**b**,**c**), 335/385 stands for pentosidine; 370/440 stands for total fluorescent AGEs.

Methylglyoxal and glyoxal, two typical α-dicarbonyl compounds, are more reactive to attacking the free amino groups of proteins than reducing sugars, and more reactive to attacking protein free amino groups than reducing sugars. Therefore, capturing methylglyoxal/glyoxal can also be an effective strategy to hinder protein glycation. As shown in Figure 5a, the capture ability of methylglyoxal and glyoxal was evaluated using HPLC analysis. After *L. fermentum* and aminoguanidine treatment for 24 h, methylglyoxal was captured by 9.6% and 34.9%, and glyoxal was captured by 20.3% and 61.3%, respectively. This showed that *L. fermentum* could capture methylglyoxal and glyoxal, and the capture of methylglyoxal was more obvious.

Furthermore, the intermediate reaction models of methylglyoxal +BSA and methylglyoxal + Arg were constructed, and *L. fermentum* was added to the culture for 4 days. The ability of *L. fermentum* to capture methylglyoxal and inhibit the intermediate stage of BSA glycation was evaluated by measuring the inhibitory activity of AGE formation during the reaction. As shown in Figure 5b, in the methylglyoxal + Arg model, the inhibition percentage of pentosidine formation was up to 52.48% and then it stabilized at approximately 38%, and the inhibition percentage of total fluorescent AGEs was up to 67.73%. After increasing the incubation time, *L. fermentum* inhibited the formation of total fluorescent AGEs. The inhibition effect gradually weakened, and the lowest value was 6.41%. In general, *L. fermentum* could prevent the binding of methylglyoxal to Arg, inhibit the formation of pentosidine and total fluorescent AGEs, and had the strongest inhibitory effect on pentosidine, which was different from the BSA + Glu model results.

As shown in Figure 5c, in the methylglyoxal + BSA model, the inhibition percentage of pentosidine formation was up to 79.39%, and the inhibition percentage of total fluorescent AGE formation was up to 96%. The inhibitory effect of glycosides was the strongest, which was consistent with the results of the methylglyoxal + Arg model.

### 3.6. Antioxidant Activity Assay

**Figure 6:**
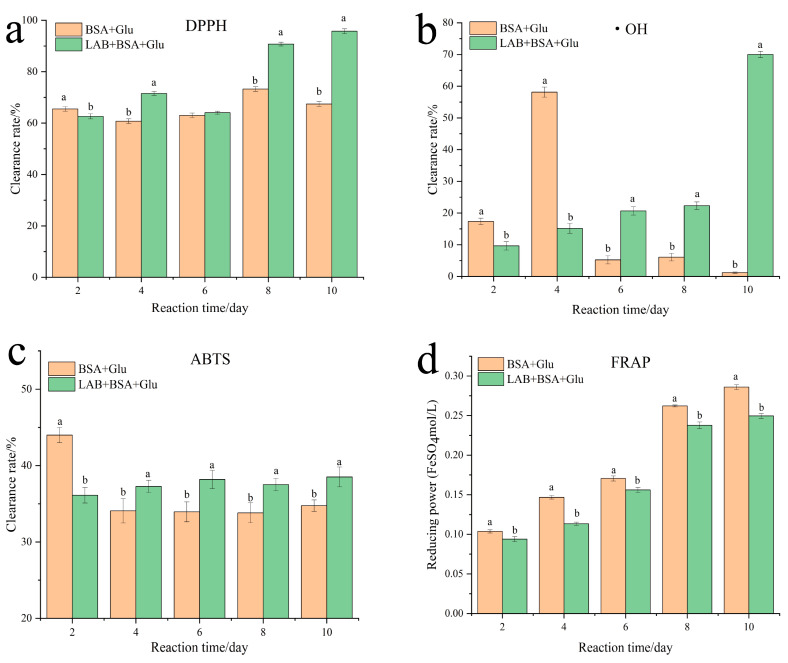
Effect of *L. fermentum* on the antioxidant activity of the BSA + Glu system. (**a**) DPPH clearance ability, (**b**) hydroxyl free radicals clearance ability, (**c**) ABTS clearance ability, and (**d**) ferric ion-reducing antioxidant power. Three replicates were performed, and different letters indicate significant differences (*p* < 0.05). Different letters indicated significant differences (*p* < 0.05).

The nonenzymatic glycation process has a large amount of intermediate free radicals; therefore, the antioxidant effect also plays an important role in preventing the protein glycation reaction. The DPPH, hydroxyl, ABTS radical scavenging capacity, and total reducing capacity were measured to evaluate the change in antioxidant activity of BSA + Glu after adding *L. fermentum*. *L. fermentum* enhanced the free radical scavenging activities of DPPH, hydroxyl, and ABTS; especially, the scavenging ability of DPPH and hydroxyl free radicals were the strongest (Figure 6a,b). After the 10 days reaction time, the DPPH and hydroxyl free radicals of LAB + BSA + Glu increased significantly by 28.32% and 68.8%, respectively, compared with those of BSA + Glu. It was shown that *L. fermentum* produced a large amount of antioxidant active substances in BSA + Glu to help scavenge free radicals in the protein glycation system, which may largely resist protein glycation. The results were positively correlated with the anti-AGE ability, but not with the total reducing ability (Figure 6d), which may indicate that the anti-AGE activity was related to the antioxidant active compounds with free radical scavenging activity, but not to the compounds with reducing ability. It may also be attributed to the enhanced antioxidant activity of *Lactobacillus* related to phenolic components [21]. Therefore, the change in total phenolic content in BSA + Glu after the addition of *L. fermentum* was further analyzed. As shown in Table 1, during the glycation reaction, *L. fermentum* slowly increased the total phenolic content, which was significantly higher than that of BSA + Glu on days 8 and 10 (*p* < 0.05).

### 3.7. The Survival of LAB in the Glycation Model

**Figure 7:**
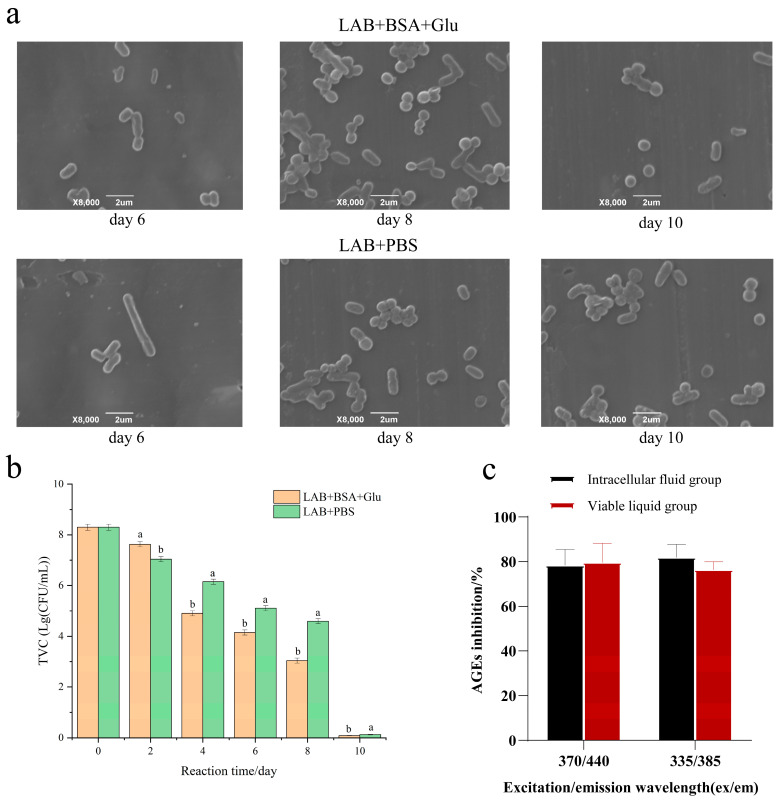
The effect of autolysis. (**a**) Morphological changes; (**b**) the total viable count (TVC) of *L. fermentum*; (**c**) anti-AGE activity of intracellular substances. Significance is at the 0.05 level (*p* < 0.05). Different letters indicated significant differences (*p* < 0.05).

As shown in Figure 7b, in the BSA + Glu glycation group and PBS control group, the initial TVC of *L. fermentum* was 2 × 10^8^ log CFU/mL. The TVC of each group gradually decreased with the increase in reaction time, and the TVC of BSA + Glu was significantly lower than that of the control group. As shown in Figure 7a, compared with the control group, the *L. fermentum* in the BSA + Glu group showed cell wall lysis and autolysis earlier. Possibly, as described by Dominika et al. [48], protein glycation products larger than 10 kDa can have a large effect on the growth of beneficial bacteria, resulting in a reduction in the number of viable bacteria. Because the molecular weight of BSA itself is 66.5 kDa, the glycation reaction further promoted the formation of BSA macromolecular aggregates, which changed the structure and functionality of BSA, resulting in the reduction in TVC of *L. fermentum*. It is also possible that *L. fermentum* was subjected to environmental stress in the protein glycation reaction system, resulting in oxidative stress and accelerated autolysis.

To study the inhibitory effect of intracellular substances of *L. fermentum* on fluorescent AGEs, we compared the inhibition rate of fluorescent AGEs after 10 days of BSA + Glu culture. Figure 7c shows that the intracellular contents of *L. fermentum* also inhibit fluorescent AGEs. This further indicates that the more the apoptotic cells, the more the intracellular material elution of *L. fermentum*. Thus, the autolysis of *L. fermentum* has a better inhibitory effect on protein glycation.

### 3.8. The BSA Structure Analysis

**Figure 8:**
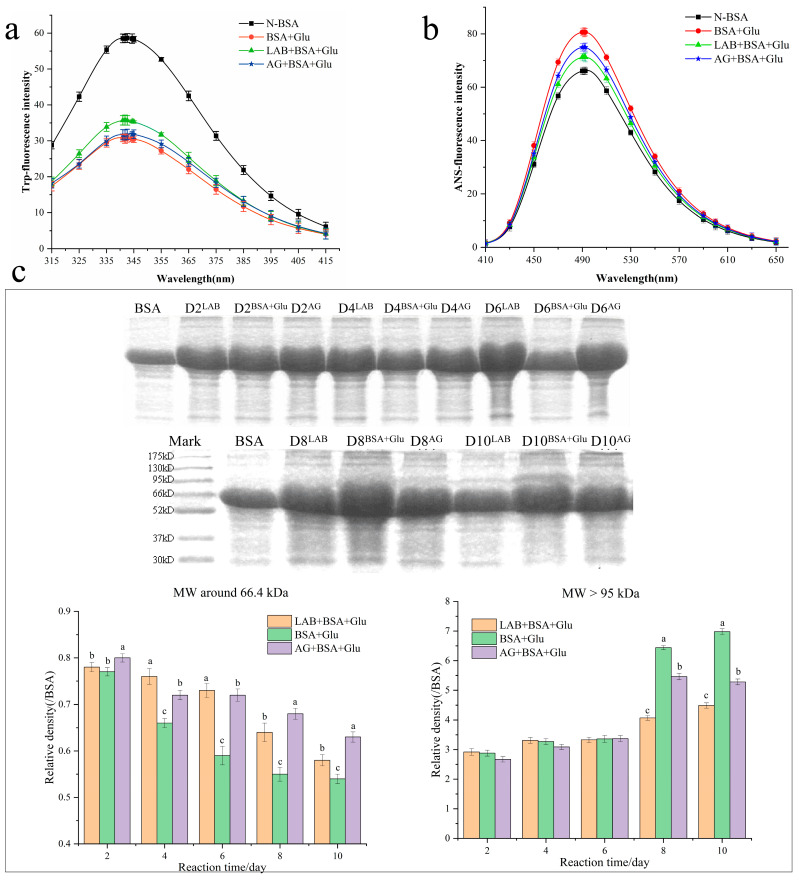
Protection of protein structure. (**a**) Trp-fluorescence spectroscopic analysis, (**b**) ANS fluorescence spectroscopic analysis, (**c**) the effect of *L. fermentum* on the relative molecular weight of glycated BSA by SDS-PAGE analysis. Significance is at the 0.05 level (*p* < 0.05). D2^LAB^ represents the protein band on day 2 of LAB + BSA + Glu; D2^BSA+Glu^ represents the protein band of BSA + Glu on day 2; D2^AG^ represents the protein band on day 2 of AG + BSA + Glu. Different letters indicated significant differences (*p* < 0.05).

Fluorescence spectroscopic analysis (Figure 8a) revealed that at an excitation wavelength of 295 nm, the maximum fluorescence intensity of the N-BSA tryptophan residue was at an emission wavelength of 342.6 nm. After 10 days of glycation reaction, the maximum emission wavelength of BSA + Glu was 341.0 nm. The blue shift effect is not significant, but and the fluorescence intensity significantly decreased (*p* < 0.05), indicating the influence of LAB on the connection or interaction between glucose hydroxyl groups and tryptophan residues, resulting in a certain shielding effect and reducing the total fluorescence value. Changes in the tryptophan microenvironment may lead to impaired BSA function, compared with BSA + Glu. The fluorescence intensity of tryptophan began to increase in the presence of *L. fermentum* and aminoguanidine, implying that *L. fermentum* had a protective effect on the conformational change induced by glucose glycation, and *L. fermentum* showed a stronger protective effect than aminoguanidine.

The surface hydrophobicity of the glycated BSA was determined using the ANS fluorescence method, both as an auxiliary assay for BSA self-aggregation and to determine the stability of the tertiary structure of BSA. Compared with N-BSA (Figure 8b), the ANS fluorescence intensity of BSA + Glu increased significantly (*p* < 0.05) after the glycation reaction. However, *L. fermentum* and aminoguanidine can reduce the damage to the BSA structure and maintain its stability.

Using SDS-PAGE analysis of N-BSA, BSA + Glu, LAB + BSA + Glu, and AG + BSA + Glu, the effect of *L. fermentum* on the relative molecular weight of glycated BSA was further explored. Among them, the BSA initial concentration was consistent. As shown in Figure 8c, N-BSA had a clear and dense protein band at approximately 66 kDa, which was consistent with the relative molecular mass of BSA (66.4 kDa). Observing the lanes on the 8th and 10th days, glycated BSA showed polydisperse bands with relative molecular weights close to 95 kDa and higher than 130 kDa on the top of the separating gel, and the color of the bands was much darker than that of N-BSA, indicating that glycation induced BSA to form cross-linked or high-molecular-weight protein aggregates. The addition of *L. fermentum* to BSA + Glu reduced the intensity of protein bands of 95 kDa and above (*p* < 0.05), and at the same tested concentrations, the inhibitory effect on the BSA + Glu band was stronger than that of aminoguanidine. Wu et al. [49] found that epicatechin gallate (ECG) inhibited the formation of AGEs more strongly than AGEs in the BSA-fructose model. The cross-linked structure of BSA was characterized using SDS-PAGE, and the results showed that with the increase in ECG concentration from 8.9 to 44.2 µg/mL, the contents of proteins with molecular weights ranging from 55 to 70 kDa and 100 to 250 kDa gradually increased and decreased, respectively. The inhibitory effect of aminoguanidine treatment at a useful concentration was significantly weaker than that of ECG, which could alleviate the cross-linking structure induced by glycation reaction. Therefore, it can be concluded that *L. fermentum* may alleviate the glycation-induced BSA cross-linked structure formation.

The degree of resistance of *L. fermentum* to changes in protein secondary structure was observed in FTIR of glycated BSA samples. The amide I band (1600–1700) of BSA was processed using Fourier self-deconvolution, second derivative, and curve fitting techniques. The peaks of the protein secondary structure are identified as follows: 1600–1639 cm^−1^ is the β-sheet structure, 1640–1650 cm^−1^ is the random coil structure, 1651–1660 cm^−1^ is the α-helix structure, and 1661–1700 cm^−1^ is the β-turn structure. The effects of N-BSA, BSA + Glu, and inhibitors on the relative content of glycated BSA secondary structure are shown in Table 2. Compared with N-BSA, the β-sheet structure reduced significantly (*p* < 0.05) in BSA + Glu (−3.83%); however, the reduction of the β-sheet structure caused by glycation was inhibited by both *L. fermentum* (−0.71%) and aminoguanidine (−2.11%). Similarly, the β-turn structure increased significantly (*p* < 0.05) in BSA + Glu (+3.1%), and glycation-induced changes in the β-turn structure were minimized by both *L. fermentum* (+1.44%) and aminoguanidine (+0.48%) inhibitors. The relative contents of the α-helix and random coil structures of BSA were also slightly altered by glycation, which was similarly inhibited to some extent by both *L. fermentum* and aminoguanidine. In experiments on BSA glycation resistance [30], aminoguanidine was significantly resistant to glycation-induced changes in the secondary structure. Therefore, the inhibition of BSA conformational changes by the addition of *L. fermentum* helped to maintain the integrity and function of the protein.

### 3.9. Effect of Active Components of L. fermentum on Glycation

**Figure 9:**
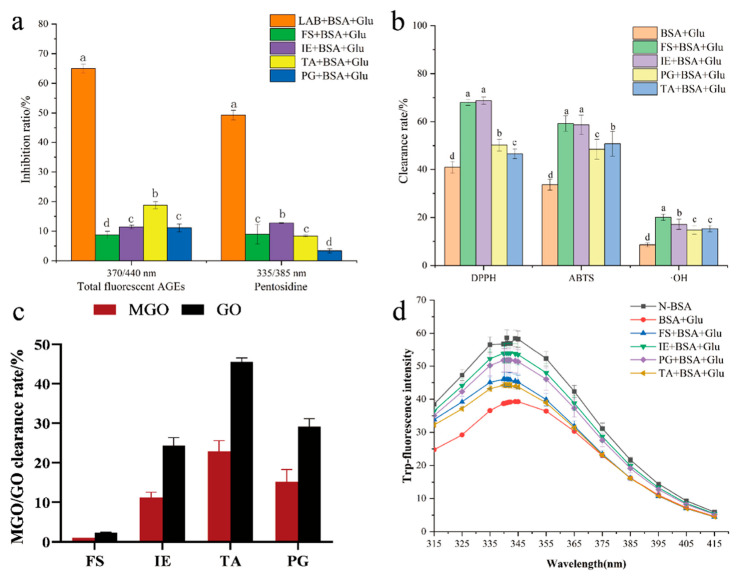
Antiglycation of *L. fermentum* active ingredients. (**a**) Inhibition of AGEs; (**b**) antioxidant activity; (**c**) capturing ability of MGO/GO; (**d**) fluorescence spectrum of tryptophan. Significance is at the 0.05 level (*p* < 0.05). FS refers to the fermentation supernatant; IE refers to the intracellular extracts; TA refers to teichoic acid; and PG refers to peptidoglycan. Different letters indicated significant differences (*p* < 0.05).

The effect of different active components of *L. fermentum* on the formation of AGEs is shown in Figure 9a. The four active components collected from *L. fermentum* with the same concentration were added to the BSA + Glu model by equal volume, and all they showed inhibitory activities. The inhibitory rates of FS, IE, TA, and PG were 8.71%, 11.46%, 18.8%, and 11.12%, respectively. Recently, active ingredients from LAB have been found to have antisaccharification and antiaging activities and to adsorb macromolecules. Thus, the antioxidant activity, the capture ability of dicarbonyl compounds, and the protection ability of the BSA structure were tested to explore the main active components of *L. fermentum* inhibiting the protein glycation reaction. The results showed that FS and IE had the highest antioxidant activity (Figure 9b); the mean scavenging rate of DPPH and ABTS was 63.58% and the mean scavenging rate of ·OH radicals was 18.12%. In the determination of the methylglyoxal/glyoxal capturing ability of the four active ingredients (Figure 9c), TA showed significant capturing ability of methylglyoxal and glyoxal, which were 22.03% and 45.58%, respectively. In the fluorescence spectra of tryptophan (Figure 9d), it was found that these four active ingredients had protective effects on the structure of BSA, in which IE had the greatest effect, followed by PG.

## 4. Conclusions

In this study, the inhibitory effect of *Lactobacillus* spp. on the nonenzymatic glycation of proteins and the formation of fluorescent AGEs were investigated. The results showed that under the environmental stress of the BSA + Glu glycation model in vitro, the autolysis rate of *L. fermentum* accelerated, the formation of fluorescent AGEs was strongly inhibited, and the formation of fructosamine and dicarbonyl compounds was inhibited. The inhibitory effect was higher than that of AG. In addition, *L. fermentum* can protect proteins from structural modifications during glycation and preserve the stability of intrinsic fluorophores. The main inhibitory mechanism against AGEs can be summarized as capturing dicarbonyl compounds, protecting protein sulfhydryl groups, inhibiting protein carbonylation, preventing the combination of glucose and amino groups, scavenging free radicals, and releasing intracellular antioxidant substances. In general, the inhibition of *L. fermentum* on glycation mainly occurs through antioxidation, the capture of dicarbonyl compounds, and the protection of the BSA structure. Among them, the antioxidant activity mainly depends on the antioxidant activity of the fermentation supernatant and intracellular extracts. Teichoic acid has the strongest trapping ability towards dicarbonyl compounds. The protection of the BSA structure depends on the peptidoglycan and intracellular extracts. Therefore, *Lactobacillus* spp., as a valuable functional food supplement, is an attractive dietary strategy to inhibit protein glycation and AGEs.

## Figures and Tables

**Table 1:** Total phenolic content of BSA + Glu and BSA + Glu incubated with aminoguanidine (AG) and *L. fermentum*.

Reaction Time/Day	BSA + Glu	LAB + BSA + Glu	AG + BSA + Glu
2	0.181 ± 0.0015 ^a^	0.165 ± 0.0025 ^b^	0.187 ± 0.0015 ^a^
4	0.179 ± 0.0010 ^a^	0.186 ± 0.0010 ^a^	0.177 ± 0.0035 ^a^
6	0.202 ± 0.0015 ^b^	0.204 ± 0.0017 ^b^	0.219 ± 0.0035 ^a^
8	0.181 ± 0.0015 ^b^	0.2 ± 0.0015 ^a^	0.199 ± 0.0036 ^a^
10	0.18 ± 0.0014 ^b^	0.198 ± 0.0100 ^a^	0.202 ± 0.0015 ^a^

Total phenolic content in µg GAE/mL. Each sample was read in triplicate. The data are presented as the mean ± standard deviation. Different letters in the same row indicated significant differences (*p* < 0.05).

**Table 2:** Secondary structure composition of N-BSA, BSA + Glu, and BSA + Glu incubated with AG and *L. fermentum*.

Conformation	N-BSA	BSA + Glu	LAB + BSA + Glu	AG + BSA + Glu
β-sheet	37.23 ± 0.03 ^a^	33.40 ± 0.04 ^c^ (−3.83%)	36.52 ± 0.01 ^a^ (−0.71%)	35.12 ± 0.002 ^b^ (−2.11%)
Random coil	20.11 ± 0.02 ^a^	19.71 ± 0.04 ^b^ (−0.4%)	19.60 ± 0.01 ^b^ (−0.51%)	20.35 ± 0.0001 ^a^ (+0.24%)
α-helix	19.55 ± 0.05 ^b^	20.69 ± 0.02 ^a^ (+1.14%)	19.75 ± 0.004 ^b^ (+0.2%)	19.95 ± 0.005 ^b^ (+0.4%)
β-turn	23.10 ± 0.04 ^c^	26.20 ± 0.01 ^a^ (+3.1%)	24.54 ± 0.03 ^b^ (+1.44%)	23.58 ± 0.01 ^c^ (+0.48%)

The values are given as percentages. Each sample was read in triplicate. The data are presented as the mean ± standard deviation. Values in parentheses represent the percentage change in the secondary structure from N-BSA. Percentage decreases and increases are denoted by “−” and “+” signs. Different letters indicated significant differences (*p* < 0.05).

## Data Availability

The original contributions presented in the study are included in the article, further inquiries can be directed to the corresponding author.
